# Identification of factor XI deficiency in Holstein cattle in Turkey

**DOI:** 10.1186/1751-0147-51-5

**Published:** 2009-01-22

**Authors:** Hasan Meydan, Mehmet A Yildiz, Fulya Özdil, Yasemin Gedik, Ceyhan Özbeyaz

**Affiliations:** 1 Ankara University, Faculty of Agriculture, Animal Sciences, 06110 Ankara, Turkey; 2 Selçuk University, Faculty of Agriculture, Animal Sciences, 42075, Konya, Turkey; 3 Ankara University, Faculty of Veterinary Medicine, 06110, Ankara, Turkey

## Abstract

**Background:**

Factor XI (*FXI*) is a plasma protein that participates in the formation of blood clots. Factor XI deficiency is autosomal recessive hereditary disorder that may be associated with excess bleeding in Holstein cattle.

**Methods:**

In this study, 225 Holstein cows reared in Turkey were screened in order to identify *FXI *genotypes. DNA extractions were obtained from the fresh blood of the cows. Amplicons of *FXI *exon 12 were obtained by Polymerase Chain Reaction (PCR), and analyzed by 2% agarose gel electrophoresis stained with ethidium bromide. Additionally, all cows were confirmed by DNA sequencing to determine whether or not there was a mutant allele.

**Results:**

Carriers of the *FXI *deficiency have two DNA fragments of 320 bp and 244 bp in size. The results of our study demonstrated that only four out of the 225 Holstein cows tested in Turkey carried the *FXI *deficiency. The frequency of the mutant *FXI *allele and the prevalence of heterozygous cows were found as 0.9% and 1.8%, respectively.

**Conclusion:**

The DNA-based test determines all genotypes, regardless of phenotype or *FXI *activity. The mutation responsible for the *FXI *deficiency had not been detected in Holstein cattle in Turkey before prior to this study. The frequency of the mutant *FXI *allele needs to be confirmed by carrying out further analyses on cattle in Turkey and the selection programs should be developed to eliminate this genetic disorder.

## Background

Factor XI is one of more than a dozen proteins involved in blood clotting. *FXI *deficiency has been identified in several species of mammals, including humans, dogs and cattle [1-4]. In cattle, *FXI *deficiency has been described in Holstein cattle in Ohio [5] and later in Canadian cattle [6], while some cases of hemorrhagic problems in British cattle have been reported [7]. *FXI *deficiency may result in prolonged bleeding and anemia. Continued bleeding from the umbilical cord is sometimes seen in affected calves. Prolonged oozing of blood following dehorning or castration may also be observed.

Affected cows frequently have pink-colored colostrum. Blood in the milk led to the identification of the condition in a British dairy herd [8]. Additionally, *FXI *deficiency causes to reduced reproductive performance and affected animals appear to be more susceptible to diseases such as pneumonia, mastitis and metritis. Therefore, the presence of this genetic defect may have a significant economic impact on the dairy industry [3,8-10]. Affected animals can survive for years with no overt clinical signs, even though they appear to have a higher mortality and morbidity rate.

Pedigree analysis indicates that *FXI *deficiency is an autosomal recessive disorder like *BLAD *(Bovine Leukocyte Adhesion Deficiency), *DUMPS *(Deficiency of Uridine Monophosphate Synthase), and *CVM *(Complex Vertebral Malformation). Accordingly, carriers (heterozygous) of the defective gene are outwardly normal, while affected animals (homozygous) have a mild hemophilia-like disorder; 25 percent of the offspring of a carrier bull and a carrier cow will be affected with a *FXI *deficiency [8]. Carrier cattle exhibit varying symptoms and degrees of reduced *FXI *activity. Current testing methods measure the activated partial thromboplastin time (APTT) to monitor *FXI *activity [6]. Although affected animals with *FXI *deficiency are relatively easy to classify, carriers of the disorder are often difficult to distinguish from normal individuals because of the overlap of activity ranges. To effectively control the spread of recessive defects such as *BLAD*, *DUMPS*, *CVM*, and *FXI *deficiency it is important to accurately identify animals that may appear clinically normal, but carry the mutant allele.

Marron et al. (2004) have identified the causative mutation for *FXI *deficiency. The authors found that the mutation consists of a 76 bp segment (AT(A)_28_TAAAG(A)_26_GGAAATAATAATTCA) insertion into exon 12 of *FXI *on chromosome 27. The insertion consists of long strings of adenine (A) bases and contains a stop codon that prevents the full-length protein from being made [9,11].

The purpose of this study was to identify and calculate the frequency of the mutant *FXI *allele in Holstein cattle reared in Turkey.

## Methods

### Samples and DNA extraction

Two hundred twenty-five Holstein cows were sampled. The blood samples were collected from three different state farms managed by the TIGEM (General Directorate of Agricultural Enterprises) in Turkey.

Blood samples were collected from the jugular vein into EDTA-containing tubes and transported to the laboratory. They were stored at -20°C until the genomic DNA extraction which was carried out by using salting-out method [12]. The genomic DNA was stored at 4°C until use.

### PCR assay

The amplification reactions were prepared in a final volume of 20 μl containing as follows; 1 × PCR buffer, 0.2 mM dNTPs, 0.5 units *Taq *DNA polymerase, 1.5 mM MgCl_2_, 20 nmol of forward (5' CCC ACT GGC TAG GAA TCG TT 3') and reverse (5' CAA GGC AAT GTC ATA TCC AC 3') primers (GenBank accession number, AY570504) as suggested by Marron et al. (2004) and 100 ng of genomic DNA. Amplification was performed using an initial denaturation of 10 minutes at 95°C, followed by 34 cycles of 30 seconds at 95°C, 60 seconds at 55°C and 30 seconds at 72°C and a final extension of 10 minutes at 72°C. PCR products resolved by electrophoresis on 2% agarose gels following by staining with ethidium bromide in TBE buffer for 40 minutes.

### DNA sequencing

After the gel electrophoresis process, the amplicons of 320 bp and 244 bp were purified using a Qiamp Mini Kit (QIAGEN, Valencia, CA, U.S.A.). The purified samples were sequenced by a Big dye terminator chemistry on an ABI 3100-Avant DNA sequencer (Applied Biosystems, Foster City, CA, U.S.A.). The DNA sequences were analyzed using the Sequencing Analysis Software Version 3.3 (Applied Biosystems, Foster City, CA, U.S.A.).

The gene frequency of the *FXI *locus was estimated by counting the number of genes [13].

## Results and Discussion

**Figure 1:**
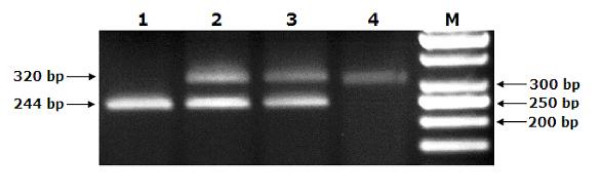
**Demonstration of all *FXI *genotypes on 2% TBE agarose gel**. Lane 1 is homozygous-unaffected producing a single 244 bp fragment, lane 2 is heterozygous (carrier) exhibiting two fragments of 244 bp and 320 bp, lane 3 is carrier control, and lane 4 is homozygous-affected control producing a single 320 bp fragment. Lane M is DNA Ladder (50 bp, Fermentase^®^). Control samples were supplied by Dr. Jonathan E. Beever.

After the PCR, the normal *FXI *allele in unaffected animals (homozygous wild type) produces a single 244 bp fragment. In homozygous affected animals, the fragment has a length of 320 bp and the heterozygous (or carrier) cattle exhibit two fragments of 244 bp and 320 bp (Fig. 1.).

Analysis of 225 Holstein cows reared in Turkey revealed that four cows were *FXI *deficiency carriers. All other cows possessed normal genotypes. The frequency of the mutant *FXI *allele and the prevalence of the carriers were calculated as 0.9% and 1.8%, respectively.

**Figure 2:**
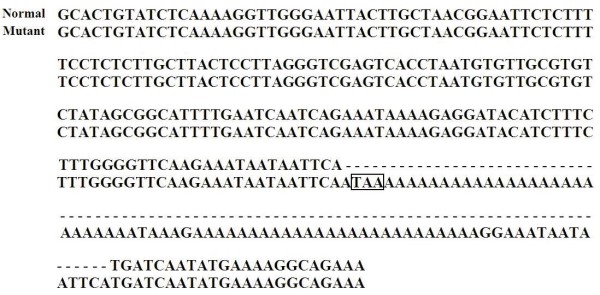
**Alignment of bovine *FXI *sequences from normal (top) and mutant (bottom) *FXI *allele**. The mutation consists of a 76 bp segment insertion into exon 12 of *FXI*. The insertion consists of long strings of adenine (A) bases and contains a stop codon. The box indicates the premature stop codon generated as a result of the insertion.

We also carried out partial sequencing in all cows in order to confirm whether these cattle were carriers or not. Our sequencing results of the mutant *FXI *allele were consistent with prior report [3] of the *FXI *gene deficiency. These results for the mutant allele revealed a mutation consisted of a 76 bp insertion containing poly Adenine sequences with a stop codon (TAA) (Fig. 2.).

It was previously hypothesized that *FXI *deficiency was due to the absence of the *FXI *protein [2]. Upon examination of the mutation in bovine *FXI*, it is most likely that the protein is not absent, but merely truncated prematurely because of the presence of a stop codon that was introduced by the insertion. Because of the truncation, the mature protein would be lacking the entire serine protease domain encoded by exons 13–15 [3].

*FXI *deficiency in cattle has been reported in many different countries, such as the USA [5], Canada [6], Britain [7], Japan [9], and the Czech Republic [4]. However, no case of *FXI *deficiency was previously reported in Holstein cattle reared in Turkey. As a first attempt, this study found that the mutant *FXI *allele frequency in Holstein cows in Turkey is 0.9% and the prevalence of carrier cattle is 1.8%. The prevalence ratio found in this study is similar to those reports in Canadian Holstein cattle (1.2%) [3], Japanese Holstein cattle (2.5%) [9], Indian Holstein cattle (0.2%) [14] and Czech Holstein and Simmental cattle (0.3%) [4].

The mutation that causes *FXI *deficiency introduces a premature stop codon. The *FXI *gene mutation in Holstein cattle reared in Turkey was confirmed by this study. Our results indicate that normal cattle have only one DNA fragment of 244 bp while heterozygous cattle exhibit two DNA fragments of 320 bp and 244 bp for the *FXI *gene deficiency.

*FXI *deficiency has been shown to adversely affect the reproductive performance of cattle; the follicular diameter of the affected cattle is small and is accompanied by lower peak estradiol concentrations in plasma near the time of ovulation. The oestrous cycle of the affected cows is characterized by reduced follicular development and a slow process of luteolysis. Reproductive performance in cattle can be affected by metritis or mastitis, since neutrophil function appears to differ in cells that were isolated from normal cattle and those that came from *FXI *deficient cattle. Additionally, it has been suggested that both homozygous and heterozygous cattle might exhibit lower calving and survival rates. Therefore, the presence of this genetic defect may have a significant economic impact on the dairy industry [9,15].

## Conclusion

The DNA-based test (PCR) described can detect the mutation responsible for *FXI *deficiency in Holstein cattle in Turkey. This is the 1st report on the FXI deficiency in Holstein cattle in Turkey. The bulls used for artificial insemination should be screened to determine whether they are FXI deficiency carriers or not. This is useful to decrease the frequency of the mutant allele in Turkish Holstein population, and selection program should be prepared to screen animals in order to eliminate the disorder.

## Competing interests

The authors declare that they have no competing interests.

## Authors' contributions

HM participated in the design of the study, collected the blood samples, carried out the extraction of genomic DNA, PCR and DNA sequencing, performed the statistical analysis and participated in the writing of the manuscript. MAY conceived of the study, participated in its design and coordination, performed the statistical analysis and participated in the writing of the manuscript. FO and YG collected the blood samples, carried out the extraction of genomic DNA, PCR and DNA sequencing, drafted the manuscript. CO participated in designing the study and drafted the manuscript. All authors read and approved the final manuscript.
